# Prevalence, Virulence, Antimicrobial Resistance, and Molecular Characterization of *Pseudomonas aeruginosa* Isolates From Drinking Water in China

**DOI:** 10.3389/fmicb.2020.544653

**Published:** 2020-12-03

**Authors:** Lei Wei, Qingping Wu, Jumei Zhang, Weipeng Guo, Qihui Gu, Huiqing Wu, Juan Wang, Tao Lei, Liang Xue, Youxiong Zhang, Xianhu Wei, Xiaocong Zeng

**Affiliations:** ^1^Guangdong Provincial Key Laboratory of Microbiology Safety and Health, State Key Laboratory of Applied Microbiology Southern China, Guangdong Institute of Microbiology, Guangdong Academy of Sciences, Guangzhou, China; ^2^School of Bioscience and Bioengineering, South China University of Technology, Guangzhou, China; ^3^College of Food Science, South China Agricultural University, Guangzhou, China; ^4^Biological Testing and Analysis Department, Guangdong Provincial Institute of Food Inspection, Guangzhou, China

**Keywords:** *Pseudomonas aeruginosa*, mineral water, spring water, virulence genes, multilocus sequence typing

## Abstract

*Pseudomonas aeruginosa* is an important opportunistic pathogen and remains a major threat to the microbial safety of drinking water. There is a lack of comprehensive data on *P*. *aeruginosa* contamination in drinking water in China. Therefore, this study aimed to determine the prevalence, genetic diversity, virulence genes, and antimicrobial resistance of *P*. *aeruginosa* isolated from mineral water and spring water in China. From January 2013 to January 2014, 314 drinking water samples were collected from 23 cities in China. Of the collected samples, 77 (24.5%) were contaminated with *P. aeruginosa*, and these comprised 34 raw water (30.4%), 39 activated carbon-filtered water (30.6%), and four final water product (3.9%). A total of 132 *P. aeruginosa* isolates were obtained, and all of them showed the presence of virulence genes, with the detection rates of *ExoU*, *ExoS*, *phzM*, *toxA*, and *lasB* genes being 7.6, 86.3, 95.5, 89.4, and 100%, respectively. All isolates were sensitive to the 14 antibiotics (ciprofloxacin, levofloxacin, ofloxacin, norfloxacin, gentamicin, tobramycin, amikacin, polymyxin B, imipenem, meropenem, aztreonam, ceftazidime, cefepime, and piperacillin/tazobactam) tested. The 132 isolates were categorized into 42 sequence types according to multilocus sequence typing, and ST235 accounted for 8.3% (11) of the total isolates. Thus, this study provides comprehensive data on the prevalence and characteristics of *P. aeruginosa* in drinking water in China and can aid in developing preventive measures against contamination during the drinking water treatment process.

## Introduction

Microbial contamination of drinking water is a common problem that has a serious impact on public health (Li et al., 2017; Wang et al., 2018). *Pseudomonas aeruginosa* is an important opportunistic pathogen and is frequently detected in drinking water (Vaz-Moreira et al., 2012; Lu et al., 2016). *P. aeruginosa* widely inhabits diverse environments, including water and soil, and is one of the main pathogens of nosocomial infections, such as cystic fibrosis (Agarwal et al., 2005; Driscoll et al., 2007). *P. aeruginosa* has a disinfectant resistance gene and can form a biofilm, which makes it the most common contaminant in drinking water production (Guerin-Mechin et al., 2000; Mah et al., 2003). Previous studies have shown that *P. aeruginosa* is the most suitable indicator of the presence of pathogens in drinking water (Mena and Gerba, 2009). The recommended international code of hygienic practice for collecting, processing, and marketing of natural mineral waters (CAC/RCP 33-1985) and on the quality of water intended for human consumption (98/83/EC) short-listed *P. aeruginosa* as a bacterial indicator of drinking water quality.

For epidemiological surveillance of *P. aeruginosa* in drinking water, molecular typing methods have the advantage of discriminatory ability and stability (Mena and Gerba, 2009). In recent studies, multilocus sequence typing (MLST), pulsed field gel electrophoresis (PFGE), enterobacterial repetitive intergenic consensus-polymerase chain reaction, and random-amplified polymorphic DNA have been conducted for tracing sources of *P. aeruginosa* (Wu et al., 2016; Bel Hadj Ahmed et al., 2019; Slekovec et al., 2019). Among these molecular typing methods, MLST and PFGE have the highest reproducibility and discriminatory ability for typing of *P. aeruginosa* isolates (De Cesare et al., 2016). Johnson et al. (2007) conducted molecular typing of 90 *P. aeruginosa* strains using PFGE and MLST, respectively, and the study proved that the resolution of PFGE and MLST was not significantly different. However, PFGE is labor time-consuming and requires sophisticated electrophoresis equipment. MLST typing is performed by comparing seven housekeeping gene sequences of bacteria. According to the result of MLST typing, the genetic diversity of bacteria is consistent in laboratories all over the world.

The pathogenicity of *P*. *aeruginosa* is mainly due to its expression of a dozen virulence factors, such as exotoxin A and pyocyanin (Feinbaum et al., 2012). The genes *ExoS* and *ExoU* encode the extracellular enzymes ExoS and ExoU, respectively. ExoS can destroy actin cytoskeleton to inhibit phagocytosis in host cells (Horna et al., 2019). ExoU is cytotoxic and has activity of phospholipid enzymes and adenopropionic acid cyclase (Luo et al., 2019). The gene *toxA* encodes exotoxin A, which can block the synthesis of cellular proteins, leading to host tissue necrosis (Wedekind et al., 2001). The gene *phzM* encodes a protein that converts phenazine-1-carboxylic acid to pyocyanin to inhibit cellular mitochondrial activity (Parsons et al., 2007). The gene *lasB* encodes elastase, which hydrolyzes host cell elastin. The synergistic effects of these various virulence factors contribute to the toxicity of *P*. *aeruginosa* (Bradbury et al., 2010).

Systematic data regarding the prevalence of *P. aeruginosa* in drinking water have not been obtained in China. In addition, information on virulent genes, antimicrobial resistance, and the genetic diversity of *P. aeruginosa* is also lacking. Therefore, in this study, we determined the prevalence, virulence genes, and antimicrobial resistance of *P. aeruginosa* in drinking water and further characterized the isolates using MLST.

## Materials and Methods

### Study Site and Sample Collection

**FIGURE 1:**
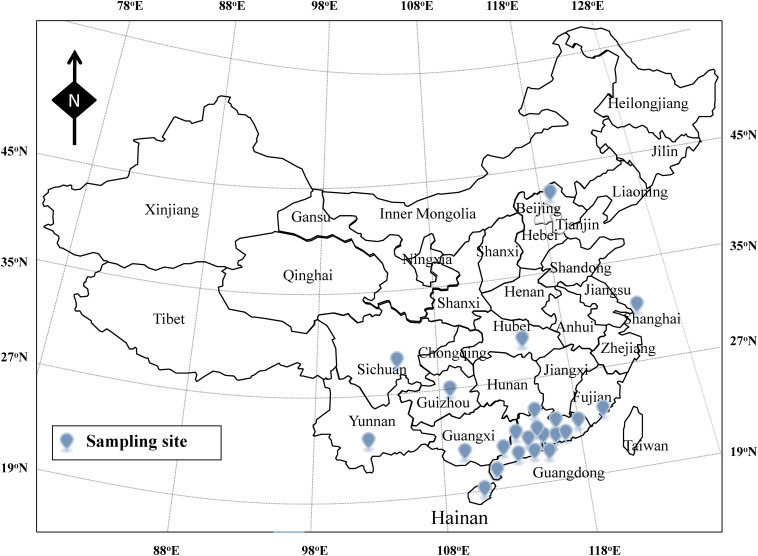
Sampling site of the drinking water in China.

**FIGURE 2:**
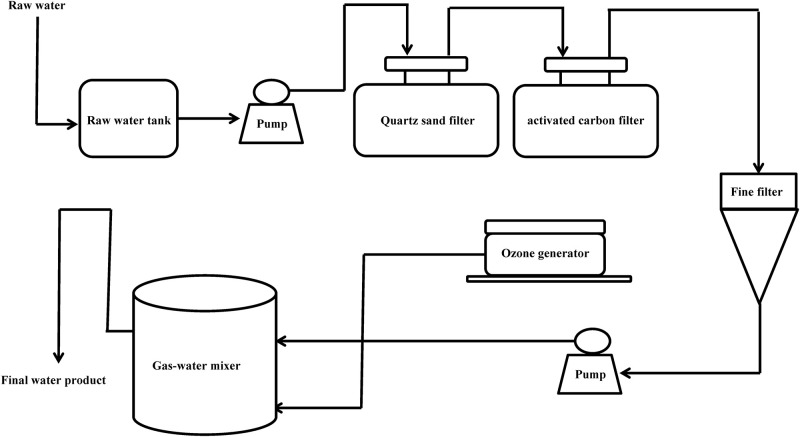
Production flow chart of mineral water and spring water in China.

From January 2013 to January 2014, according to the guideline of ISO-16266-2008, we collected water samples from 101drinking water factories (including 58 spring water and 43 mineral water factories) in 23 cities of China (Beijing, Shanghai, Chengdu, Wuhan, Xiamen, Guiyang, Kunming, Nanning, Haikou, Yangjiang, Yufu, Zhaoqing, Qingyuan, Shaoguan, Heyuan, Meizhou, Huizhou, Guangzhou, Dongguan, Foshan, Zhongshan, Zhuhai, and Jiangmen) (Figure 1). As shown in Figure 2, one part of raw water, one part of activated carbon-filtered water, and one part of final water product were collected in each drinking water factories. Since 11 drinking water factories (including seven spring water and four mineral water factories) have two raw water sources, a total of 314 water samples were collected, including 112 raw water samples, 101 activated carbon-filtered water samples, and 101 final water product samples. All samples were placed below 4°C during transportation and testing was performed immediately after receiving the samples.

### Isolation and Enumeration of *P. aeruginosa*

The detection and the enumeration of *P*. *aeruginosa* were conducted as described in ISO-16266-2008 International Organization of Standardization [ISO] (2008). Briefly, water sample (250 ml) was filtered through a 0.45-μm membrane (Millipore Co., Billerica, MA, United States) in a stainless steel multi-line filter system (Huankai Co., Guangzhou, China). The membrane was placed on CN agar medium (Huankai Co., Guangzhou, China), a selective medium for *P*. *aeruginosa*, and then cultured at 36°C for 48 h. *P. aeruginosa* colonies were identified based on any of the following: colonies with green color or producing fluorescence and ammonia in ethyl amide broth or forming red color and oxidase positive, producing ammonia in ethyl amide broth and fluorescence on King’s B medium. All *P. aeruginosa* isolates were further identified by a species-specific *ecfX* gene. Genomic DNA was extracted using a Bacterial Genomic DNA Purification kit (Dongsheng Biotech, Guangzhou, China), according to the manufacturer’s instruction. *P. aeruginosa* was identified by amplification of 200-bp fragments with primer pairs ecfX-F (5′ CCTTCCCTCCTTCCCCCCATGCCTATCAGGCGTTCCAT)/ ecfX-R (5′ CCTTCCCTCCTTCCCCCCGGCGATCTGGAAA AGAAATG) (Shi et al., 2012).

### Detection of Virulence Genes

Five virulence genes, namely, *ExoU*, *ExoS*, *phzM*, *toxA*, and *lasB*, were detected with universal primer multiplex PCR described by Shi et al. (2012). The PCR mixture (30 μl) contained 1 μl template (50 ng), 200 μmol/l dNTPs, 3 μl × 10 Buffer, 1.5 mmol/l Mg^2+^, primer mixture (0.045 μmol/l EXOU130-F/R, 0.06 μmol/l EXOS276-F/R, 0.09 μmol/l PYO366-F/R, 0.09 μmol/l ETA433-F/R, 0.045 μmol/l ELA556-F/R, and 2.0 μmol/l UP), and 5 U Taq enzyme. The amplification parameters were as follows: initial denaturation at 95°C for 5 min, 30 cycles of 30 s at 95°C, 30 s at 58°C, and 50 s at 72°C, and a final extension at 72°C for 10 min. All primers were synthesized by BGI instrument (Shenzhen, China) (Supplementary Table 1). *P. aeruginosa* CMCC10104 (Guangdong Culture Collection Centre) was used as the positive control, and distilled water was used as the negative control.

### Antibiotic Resistance

Testing the antibiotic resistance of all *P. aeruginosa* isolates was performed by the Kirby–Bauer disk diffusion method according to the Clinical and Laboratory Standards Institute (CLSI) guidelines (Clinical and Laboratory Standards Institute [CLSI], 2010). *P. aeruginosa* CMCC 10104 (Guangdong Culture Collection Centre) was tested as positive control. A panel of antibiotics at a specific concentration per disk was tested: ciprofloxacin (5 μg), levofloxacin (5 μg), ofloxacin (5 μg), norfloxacin (10 μg), gentamicin (10 μg), tobramycin (10 μg), amikacin (30 μg), polymyxin B (300 U), imipenem (10 μg), meropenem (10 μg), aztreonam (30 μg), ceftazidime (30 μg), cefepime (30 μg), and piperacillin/tazobactam (110 μg) (Oxoid Co., Hampshire, United Kingdom). All *P. aeruginosa* isolates were classified as resistant, intermediate, or sensitive using the breakpoints specified by CLSI (Supplementary Table 2).

### MLST Analysis

According to the protocols published on the *P. aeruginos*a pubMLST website,^1^ MLST analysis was performed using seven housekeeping genes, namely, *acsA*, *aroE*, *guaA*, *mutL*, *nuoD*, *ppsA*, and *trpE*, and all primers were synthesized by BGI instrument (Shenzhen, China) (Table 1). According to the experimental method described by Curran et al. (2004), the housekeeping genes were amplified for all *P. aeruginos*a isolates, and PCR products were selected and sent to BGI Instrument (Shenzhen, China) for sequencing. The gene sequences were then compared to the existing sequences on *P. aeruginos*a pubMLST website^2^ to assign allele numbers and define sequence types (STs). Then, cluster analysis was conducted using BioNumerics 7.6 software (Applied Maths, Sint-Martens-Latem, Belgium), and a minimum spanning tree was generated from the allelic profiles of the isolates.

**TABLE 1:** PCR primers of housekeeping genes.

**Primers**	**Sequence (5′→3′)**	**Base pairs**
acsA-F	ACCTGGTGTACGCCTCGCTGAC	842
acsA-R	GACATAGATGCCCTGCCCCTTGAT	
aroE-F	TGGGGCTATGACTGGAAACC	825
aroE-R	TAACCCGGTTTTGTGATTCCTACA	
guaA-F	CGGCCTCGACGTGTGGATGA	940
guaA-R	GAACGCCTGGCTGGTCTTGTGGTA	
mutL-F	CCAGATCGCCGCCGGTGAGGTG	940
mutL-R	CAGGGTGCCATAGAGGAAGTC	
nuoD-F	ACCGCCACCCGTACTG	1,042
nuoD-R	TCTCGCCCATCTTGACCA	
ppsA-F	GGTCGCTCGGTCAAGGTAGTGG	989
ppsA-R	GGGTTCTCTTCTTCCGGCTCGTAG	
trpE-F	GCGGCCCAGGGTCGTGAG	811
trpE-R	CCCGGCGCTTGTTGATGGTT	

## Results

### Contamination of *P. aeruginosa* in Drinking Water

In this study, 50 (49.5%) drinking water factories were found to be positive for *P. aeruginosa* (Supplementary Table 3). Of all the water samples (314), 77 (24.5%) were contaminated with *P. aeruginosa*, including 34 (30.4%) raw water, 39 (38.6%) activated carbon-filtered water, and four (3.9%) final water products. A total of 132 *P. aeruginosa* isolates were obtained from the 77 positive samples for *P. aeruginosa* (Supplementary Table 4). The contamination rates of the different samples are shown in Table 2. Among the 133 mineral water samples, 18 (13.5%) were positive for *P. aeruginosa*, including eight (17%) raw water and 10 (23.3%) activated carbon-filtered water. The final water product was not found to be positive for *P. aeruginosa* among the mineral water samples. Among the 181 spring water samples, 59 (32.6%) were positive for *P. aeruginosa*, including 26 (40%) raw water, 29 (50%) activated carbon-filtered water, and four (6.9%) final water product. Among the 77 contaminated samples with *P. aeruginosa* (Supplementary Table 3), the contamination level of spring water samples of raw water, activated carbon-filtered water, and final water product was 22.2, 40.2, and 77.5 colony forming units (CFU)/250 ml, respectively, compared with 4.4, 11.8, and 0 CFU/250 ml, respectively, for mineral water samples. Among the 112 raw water samples, there were 98 groundwater and 14 surface raw water samples. As shown in Table 3, 23 (23.5%) underground and six (42.9%) surface raw water samples were contaminated with *P. aeruginosa*.

**TABLE 2:** Prevalence of *Pseudomonas aeruginosa* from mineral water and spring water.

**Samples**	**Raw water**		**Activated carbon filtered water**		**Final water product**		**Total**	
	**Positive amount/total**	**Contamination rate (%)**	**Positive amount/total**	**Contamination rate (%)**	**Positive amount/total**	**Contamination rate (%)**	**Positive amount/total**	**Contamination rate (%)**
M	8/47	17.0	10/43	23.3	0/43	0	18/133	13.5
S	26/65	40.0	29/58	50.0	4/58	6.9	59/181	32.6
Average	34/112	30.4	39/101	38.6	4/101	3.9	77/314	24.5

*M and S water represent mineral and spring water, respectively.*

**TABLE 3:** Prevalence of *Pseudomonas aeruginosa* in surface water and groundwater.

**Samples**	**Positive amounts**	**Total amounts**	**Contamination rates (%)**
Surface water	6	14	42.9
Groundwater	23	98	23.5

### Detection of Virulence Genes

In this study, species-specific *ecfX* gene was found in all 132 *P. aeruginosa* isolates. The detection results for virulence genes (*ExoU*, *ExoS*, *phzM*, *toxA*, and *lasB*) in the 132 isolates are shown in Table 4. Among the isolates, 132 (100%), 126 (95.5%), 118 (89.4%), 10 (7.6%), and 114 (86.3%) were positive for *lasB*, *phzM*, *toxA*, *ExoU*, and *ExoS* genes, respectively. The virulence profiles of the isolates are shown in Table 5. Virulence profile B (*ExoS*, *phzM*, *toxA*, and *lasB*) accounted for 74.2% (*n* = 98) of all isolates, followed by virulence profile F (*ExoS*, *phzM*, and *lasB*) accounting for 7.6% (*n* = 10).

**TABLE 4:** Virulence genes of 132 *Pseudomonas aeruginosa* isolates.

**Virulence gene**	**Number of positive sample (%)**
*ExoU*	10 (7.6)
*ExoS*	114 (86.3)
*phzM*	126 (95.5)
*toxA*	118 (89.5)
*lasB*	132 (100)
*ecfX*	132 (100)

**TABLE 5:** Virulence profiles of 132 *Pseudomonas aeruginosa* isolates.

**Virulence profiles**	**Virulence gene**	**Isolate numbers**	**Number of positive samples (%)**
A	*ExoU*, *phzM*, *toxA*, *lasB*	1, 2, 31–34	6 (4.5%)
B	*ExoS*, *phzM*, *toxA*, *lasB*	3–5, 8–11, 14, 17, 18, 21–30, 35–42, 44–63, 65–73, 81–105, 110–112, 117–124, 126, 129–132	98 (74.2%)
C	*phzM*, *toxA*, *lasB*	77–80, 107–108	8 (6.0%)
D	*ExoS*, *toxA*, *lasB*	12, 13, 15, 16, 19, 20	6 (4.5%)
E	*ExoU*, *phzM*, *lasB*	106, 125, 127, 128	4 (3.0%)
F	*ExoS*, *phzM*, *lasB*	6, 7, 43, 64, 74–76, 109, 113, 114	10 (7.6%)

### Antibiotic Resistance

All 132 isolates were classified as resistant, intermediate, or sensitive according to the diameter of the inhibition zone, as described by CLSI. As shown in Supplementary Figure 1, the result of antimicrobial susceptibilities showed that all 132 isolates were sensitive to 14 antibiotics (ciprofloxacin, levofloxacin, ofloxacin, norfloxacin, gentamicin, tobramycin, amikacin, polymyxin B, imipenem, meropenem, aztreonam, ceftazidime, cefepime, and piperacillin/tazobactam). No *P. aeruginosa* isolate was resistant to the antibiotics tested.

### MLST

**FIGURE 3:**
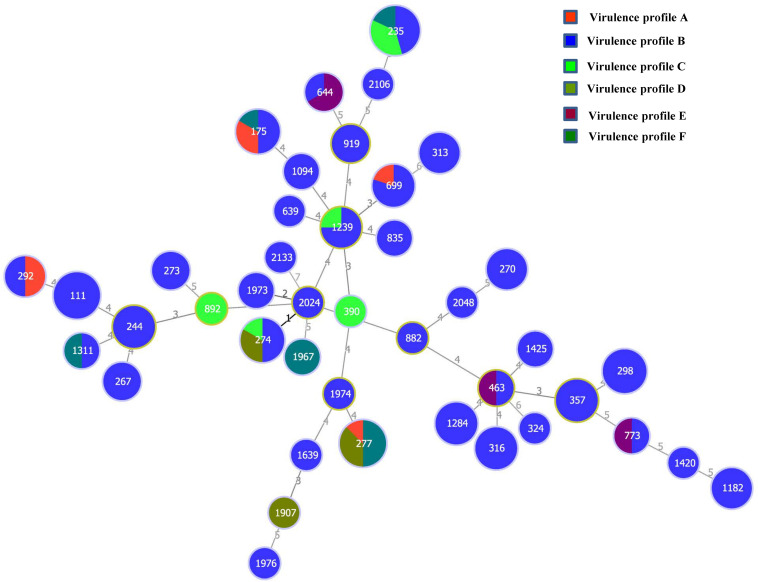
Minimum spanning tree based on multilocus sequence typing data for the 132 *P. aeruginosa* isolates examined in this study. Each circle represents one sequence type (ST). The size of the circle is related to the number of strains within this ST. The colors in the circles represent the virulence profiles.

All the 132 *P. aeruginosa* isolates from the 23 cities and different sample types were characterized by MLST analysis, which showed that the isolates could be classified into 42 STs (Supplementary Table 3). ST235 accounted for 8.3% (11) of the total isolates, followed by ST111 at 6.1% (8) and ST277 at 6.1% (8). Additionally, 14 (33.3%) of the 42 STs included a single strain, and eight (19.0%) STs included two isolates. According to the allele numbers of the seven housekeeping genes, a minimum spanning tree was generated to reveal the relationships between the 132 isolates (Figure 3). The STs of the isolates were then further analyzed relative to the sample type and virulence profiles (Figure 3 and Supplementary Figure 2). ST1420, ST1907, ST1974, ST2048, and ST2133 only existed in raw water samples. Five *P. aeruginosa* isolates from the final water product included five STs (ST175, ST267, ST298, ST324, and ST1182). Furthermore, 11 ST235 isolates included three virulence profiles (B, C, and F), eight ST277 isolates included three virulence profiles (A, B, and D), and eight ST111 isolates showed only one virulence profile (B).

## Discussion

According to the guidelines on the quality of water intended for human consumption (98/83/EC), drinking water should not test positive for *P. aeruginosa*. Although the prevalence of *P. aeruginosa* has been systematically studied in the final water product, the data on *P. aeruginosa* contamination are lacking for the whole treatment process. In our investigation, a large number of samples (112 raw water samples, 101 activated carbon-filtered water samples, and 101 final water product samples) were collected from 101 drinking water factories covering the whole treatment process, and these data can be used for risk assessment of *P. aeruginosa*. The results of this study showed that 77 (24.5%) samples were contaminated with *P. aeruginosa* among the 314 water samples. Consistent with previous investigations conducted by Wu et al. (2016), the present result showed that *P. aeruginosa* contamination of raw water (30.4%) and activated carbon-filtered water (38.6%) was significantly higher than that of raw water (3.9%). Notably, the contamination rate of surface raw water was significantly higher than that of ground raw water. Thus, groundwater is better than surface water as raw water. The main reason for this is that surface raw water is generally drained from pipes to open cistern and is exposed to air for a long time without effective protection measures. In this study, the *P. aeruginosa* contamination rate of activated carbon-filtered water was the highest among the three kinds of water sample tested. Activated carbon filter system is one of the most important processes in drinking water treatment and is used to adsorb organic pollutants and microbes to purify water (Feng et al., 2013). The *P. aeruginosa* contamination rate of activated carbon-filtered water can be used to indicate the degree of production specification. Our previous studies have shown that activated carbon filter can get enriched with microorganisms and is the most serious microbial contamination problem in the whole treatment process of drinking water (Wei et al., 2017). Therefore, manufacturers of drinking water must clean, disinfect, and replace activated carbon filters regularly. Stoler et al. (2015) reported that 41% of 80 sachet water samples were positive for *P. aeruginosa*. In this study, no *P. aeruginosa* was detected in the final water product of mineral water. However, the contamination rate of the final water product was 6.9% in the case of spring water, which is harmful to the health of the costumers. The high contamination rate of *P. aeruginosa* in spring water may be caused by the irregular production process during the manufacturing process, including inadequate disinfection, the formation of biofilms of *P. aeruginosa* in the pipeline, and improper maintenance of activated carbon filter. Hence, manufacturers of drinking water should pay attention to the control and management of the production process to ensure the safety of drinking water.

The pathogenesis of *P. aeruginosa* is closely related to the virulence genes *ExoU*, *ExoS*, *phzM*, *toxA*, and *lasB* (Mavrodi et al., 2001; Strateva et al., 2010; Shi et al., 2012). In this study, all 132 isolates carried at least three virulence genes, a fact which was in contrast to a previous study (Wu et al., 2016). Notably, 132 (100%), 126 (95.5%), 118 (89.4%), 10 (7.6%), and 114 (86.3%) isolates were positive for *lasB*, *phzM*, *toxA*, *ExoU*, and *ExoS* genes, respectively, indicating that these virulence genes are widely distributed among *P. aeruginosa*. Consistent with previous investigations, the virulence genes *ExoU* and *ExoS* were not detected in the same isolates (Wolfgang et al., 2003; Shaver and Hauser, 2004). Furthermore, among the six virulent profiles of all isolates (Table 5), virulence profile B was predominant (*ExoS*, *phzM*, *toxA*, and *lasB*), accounting for 74.2% (*n* = 98) of the isolates. Importantly, the presence of the virulence genes indicates that *P. aeruginosa* is a potential pathogenic bacterium. The virulence gene expression in *P. aeruginosa* is mainly related to quorum sensing. Therefore, future studies should be conducted to study how these virulence genes are expressed in *P. aeruginosa*. In previous studies, clinical *P. aeruginosa* was generally resistant to antibiotics (Keating et al., 2013; Munita et al., 2017). However, no *P. aeruginosa* isolate was resistant to the 14 different antibiotics tested in our study. Silva et al. (2008) isolated 30 *P. aeruginosa* strains from drinking water that were resistant to one or more antibiotics. The difference in the results may be due to the different sources of the samples. In this study, the raw water mainly came from groundwater, and the small amount of surface raw water samples was also taken from sparsely populated remote areas, with less exposure to outside influences. These environments are rarely contaminated with antibiotics, so *P. aeruginosa* has not been induced to be antibiotic resistant.

Multilocus sequence typing is a crucial epidemiological typing method and is based on the sequences of seven different housekeeping genes. It has been used in studies on the evolution and population diversity of *P. aeruginosa* isolates and is advantageous, owing to its discriminatory value and rapid *P. aeruginosa* typing (Castañeda-Montes et al., 2018; Kainuma et al., 2018). In this study, the MLST results provided a better overview of *P. aeruginosa* diversity. Of the 42 STs, 14 (33.3%) included a single strain and eight (19.0%) included two isolates, indicating the high genetic diversity of the isolates. As shown in Figure 3, a good correlation among MLST and virulence profiles was found in some isolates. However, there was no significant correlation between ST type and sample type (Supplementary Figure 2). All isolates that belonged to ST111 (*n* = 8), ST298 (*n* = 6), ST244 (*n* = 5), ST316 (*n* = 6), ST1284 (*n* = 5), and ST357 (*n* = 5) showed the same virulence profile B (*ExoS*, *phzM*, *toxA*, and *lasB*). As no antibiotic-resistant isolate was found in this study, no correlation was observed between the MLST profiles and the antibiotic resistance profiles of the isolates.

In summary, this study investigated the prevalence, virulence, antimicrobial resistance, and molecular characteristics of *P. aeruginosa* isolates from drinking water samples across different cities of China. Of the water samples tested, 24.5% were contaminated, and the contamination rate of the final water product was 6.9% in the case of spring water, suggesting that drinking water products could be a potential vehicle for the transmission of *P. aeruginosa*. In addition, since activated carbon filters, the most serious potential contamination site in the whole production process, also showed a high degree of *P. aeruginosa* contamination, manufacturers of drinking water should regularly clean, disinfect, and replace activated carbon filters. Furthermore, our data indicated that all 132 isolates carried at least three virulence genes, indicating that they may have a potentially pathogenic effect on the health of consumers. The MLST data from this study can be used to trace the possible origins of *P. aeruginosa*, which would aid in developing effective and precise prevention and control measures against *P. aeruginosa* contamination of the whole drinking water production process.

## Data Availability Statement

The original contributions presented in the study are included in the article/Supplementary Material, further inquiries can be directed to the corresponding author.

## Author Contributions

QW, JZ, and LW conceived and designed the experiments. LW, XW, and WG performed the experiments. LW, QG, and JW analyzed the data. LX, YZ, and XZ contributed reagents, materials, and analysis tools. LW, HW, and TL contributed to the writing of the manuscript. All authors contributed to the article and approved the submitted version.

## Conflict of Interest

The authors declare that the research was conducted in the absence of any commercial or financial relationships that could be construed as a potential conflict of interest.

## Footnotes

1 http://pubmlst.org/paeruginosa/

2 www.pubmlst.org/paeruginosa
