# Supplementary material for: Prevalence, Virulence, Antimicrobial Resistance, and Molecular Characterization of Pseudomonas aeruginosa Isolates From Drinking Water in China
Source: Front Microbiol. 2020 Dec 3;11:544653. doi: 10.3389/fmicb.2020.544653 (PMC7744469; doi:10.3389/fmicb.2020.544653)
Supplement: Supplementary file 1 [file Data_Sheet_1.docx]

Supplementary Material

**Prevalence, virulence, antimicrobial resistance and molecular characterization of *pseudomonas aeruginosa* isolates from drinking water in China**

**Lei Wei^1,2^, Qingping Wu^2*^, Jumei Zhang^2^, Weipeng Guo^2^, Qihui Gu^2^, Huiqing Wu^2^, Juan Wang^3^, Tao Lei^2^,Liang Xve^2^, Youxiong Zhang^2^, Xianhu Wei^2^, Huiqing Wu^2^, Xiaocong Zeng^4^**

*** Correspondence:** Qingping Wu: E-mail: [wuqp203@163.com](mailto:wuqp203@163.com)

## 1. Supplementary Table

Tabel S1 PCR primers for virulence genes detection

| Primers | Sequence (5’→3’) | bp |
| --- | --- | --- |
| ecfX-F | CCTTCCCTCCTTCCCCCCATGCCTATCAGGCGTTCCAT | 200 |
| ecfX-R | CCTTCCCTCCTTCCCCCCGGCGATCTGGAAAAGAAATG |  |
| ExoU-F | CCTTCCCTCCTTCCCCCCCCAACACATTAGCAGCGAGA | 130 |
| ExoU-R | CCTTCCCTCCTTCCCCCCTGGGAGTACATTGAGCAGCA |  |
| ExoS-F | CCTTCCCTCCTTCCCCCCCATCCTCAGGCGTACATCCT | 276 |
| ExoS-R | CCTTCCCTCCTTCCCCCCATCGATGTCAGCGGGATATC |  |
| phzM-F | CCTTCCCTCCTTCCCCCCCGGCGAAGACTTCTACAGCT | 366 |
| phzM-R | CCTTCCCTCCTTCCCCCCAGGTAGATATCGCCGTTGGA |  |
| toxA-F | CCTTCCCTCCTTCCCCCCATGGTGTAGATCGGCGACAT | 433 |
| toxA-R | CCTTCCCTCCTTCCCCCCAAGCCTTCGACCTCTGGAAC |  |
| lasB-F | CCTTCCCTCCTTCCCCCCACATCGCCCAACTGGTCTAC | 556 |
| lasB-R | CCTTCCCTCCTTCCCCCCACCAGCGGATAGAACATGGT |  |
| UP | CCTTCCCTCCTTCCCCCC | - |

Tabel S2 Performance Criteria for detection of antibiotic sensitivity of Pseudomonas aeruginosa

| **Antibiotics** | **Sensitive (mm)** | **Intermediate (mm)** | **Resistant (mm)** |
| --- | --- | --- | --- |
| Ciprofloxacin (5 µg) | ≥21 | 16-20 | ≤15 |
| Levofloxacin (5 µg) | ≥17 | 14-16 | ≤13 |
| Ofloxacin (5 µg) | ≥16 | 13-15 | ≤12 |
| Norfloxacin (10 µg) | ≥17 | 13-16 | ≤12 |
| Gentamicin (10 µg) | ≥15 | 13-14 | ≤12 |
| Tobramycin (10 µg) | ≥15 | 13-14 | ≤12 |
| Amikacin (30 µg) | ≥17 | 15-16 | ≤14 |
| Polymyxin B (300 U) | ≥12 | - | ≤11 |
| Imipenem (10 µg) | ≥16 | 14-15 | ≤13 |
| Meropenem (10 µg) | ≥16 | 14-15 | ≤13 |
| Aztreonam (30 µg) | ≥22 | 16-21 | ≤15 |
| Ceftazidime (30 µg) | ≥18 | 15-17 | ≤14 |
| Cefepime (30 µg) | ≥18 | 15-17 | ≤14 |
| Piperacillin/tazobactam (110 µg) | ≥18 | - | ≤17 |

Tabel S3 Detection results of *Pseudomonas aeruginosa* in drinking water factories

| ManufactoryNo. | Raw water **(CFU/250 ml)** | Activated Carbon FilteredWater (CFU/250 ml) | Final Water product **(CFU/250 ml)** |
| --- | --- | --- | --- |
| NN-S1 |  | 40.0 |  |
| NN-S2 | 1.0 | 2.0 |  |
| NN-M3 | 7.0 ^S^ | 3.0 |  |
| XM-S1 |  | 20.0 |  |
| XM-S2 |  | 2.0 |  |
| KM-S2 | 1.0 |  |  |
| GY-S1 | 30.0 | 20.0 |  |
| GY-S2 | 1.0 | 3.0 |  |
| WH-S1 | 3.0 ^S^ | 2.0 |  |
| WH-S2 |  | 1.0 |  |
| WH-M3 |  | 1.0 |  |
| CD-S1 | 2.0 | 2.0 |  |
| CD-S2 |  | 2.0 |  |
| HK-S1 | 5.0 | 3.0 |  |
| HK-S2 | 4.0 | 4.0 |  |
| HK-M3 | 1.0 |  |  |
| HK-M4 | 3.0 |  |  |
| SH-S1 | 5.0 | 2.0 |  |
| SH-S1 | 4.0 |  |  |
| BJ-S1 | 3.0 | 3.0 | 1.0 |
| BJ-S2 | 2.0 | 3.0 |  |
| BJ-M3 |  | 2.0 |  |
| GZ-S2 | 3 | 1 |  |
| GZ-M3 |  | 1 |  |
| ZS-M5 | 1 | 8 |  |
| HZ-S13 | ＞300 | 3 | ＞300 |
| JM-M15 | 4 13 | - |  |
| GZ-S16 | 4 | - | 3 |
| FS-S23 |  | 1 |  |
| FS-S25 | 19^S^, 8^S^ | 15 |  |
| JM-S30 | 9^S^ | - |  |
| DG-M31 |  | 6 |  |
| DG-M32 |  | 17 |  |
| ZH-S33 | 1 | ＞300 |  |
| ZH-M35 | 1 | 4 |  |
| GZ-S39 | 35 | 14 |  |
| GZ-S42 | 76 | ＞300 | 5 |
| QY-S4 | 2^S^ |  |  |
| SG-S7 | 35, 15 | 67 |  |
| MZ-M1 |  | 29 |  |
| MZ-S7 |  | 1 |  |
| HY-S8 |  | 4 |  |
| HY-S9 |  | 50 |  |
| YJ-S1 |  | 1 |  |
| YF-S4 | 1 |  |  |
| YF-S5 | 1 |  |  |
| YJ-S6 |  | 1 |  |
| YJ-S7 |  | ＞300 |  |
| ZQ-M9 | 5 | 47 |  |

Tabel S4 Information of *Pseudomonas aeruginosa isolates*

| Isolates No. | Sample No. | source | Location | ST | Virulence profiles |
| --- | --- | --- | --- | --- | --- |
| 1 | SH-S1-1 | Raw water | Shanghai | 175 | A |
| 2 | SH-S1-1 | Raw water | Shanghai | 175 | A |
| 3 | SH-S1-2 | Activated carbon filtered water | Shanghai | 175 | B |
| 4 | SH-S1-2 | Activated carbon filtered water | Shanghai | 235 | B |
| 5 | SH-S2-1 | Raw water | Shanghai | 316 | B |
| 6 | WH-S1-1 | Raw water | Wuhan | 277 | F |
| 7 | WH-S1-1 | Raw water | Wuhan | 277 | F |
| 8 | WH-S1-2 | Activated carbon filtered water | Wuhan | 1284 | B |
| 9 | WH-S1-2 | Activated carbon filtered water | Wuhan | 1284 | B |
| 10 | WH-S2-2 | Activated carbon filtered water | Wuhan | 919 | B |
| 11 | WH-M3-2 | Activated carbon filtered water | Wuhan | 1973 | B |
| 12 | HK-S1-1 | Raw water | Haikou | 274 | D |
| 13 | HK-S1-2 | Activated carbon filtered water | Haikou | 274 | D |
| 14 | HK-S1-2 | Activated carbon filtered water | Haikou | 235 | B |
| 15 | HK-S2-1 | Raw water | Haikou | 277 | D |
| 16 | HK-S2-2 | Activated carbon filtered water | Haikou | 277 | D |
| 17 | HK-S2-2 | Activated carbon filtered water | Haikou | 1311 | B |
| 18 | HK-M3-1 | Raw water | Haikou | 1420 | B |
| 19 | HK-M4-1 | Raw water | Haikou | 1907 | D |
| 20 | GY-S1-1 | Raw water | Guiyang | 277 | D |
| 21 | GY-S1-2 | Activated carbon filtered water | Guiyang | 1425 | B |
| 22 | GY-S2-1 | Raw water | Guiyang | 1425 | B |
| 23 | GY-S2-2 | Activated carbon filtered water | Guiyang | 919 | B |
| 24 | NN-S1-2 | Activated carbon filtered water | Nanning | 244 | B |
| 25 | NN-S2-1 | Activated carbon filtered water | Nanning | 244 | B |
| 26 | NN-S2-2 | Raw water | Nanning | 292 | B |
| 27 | NN-M3-1 | Activated carbon filtered water | Nanning | 292 | B |
| 28 | KM-M3-2 | Raw water | Nanning | 244 | B |
| 29 | KM-S2-1 | Activated carbon filtered water | Kunming | 699 | B |
| 30 | CD-S1-1 | Raw water | Chengdu | 699 | B |
| 31 | CD-S1-2 | Activated carbon filtered water | Chengdu | 699 | A |
| 32 | CD-S1-2 | Activated carbon filtered water | Chengdu | 277 | A |
| 33 | CD-S2-2 | Activated carbon filtered water | Chengdu | 292 | A |
| 34 | CD-S2-2 | Activated carbon filtered water | Chengdu | 292 | A |
| 35 | XM-S1-2 | Activated carbon filtered water | Xiamen | 1284 | B |
| 36 | XM-S1-2 | Activated carbon filtered water | Xiamen | 1284 | B |
| 37 | XM-S1-2 | Activated carbon filtered water | Xiamen | 316 | B |
| 38 | XM-S1-2 | Activated carbon filtered water | Xiamen | 270 | B |
| 39 | XM-S1-2 | Activated carbon filtered water | Xiamen | 270 | B |
| 40 | XM-S1-2 | Activated carbon filtered water | Xiamen | 270 | B |
| 41 | XM-S1-2 | Activated carbon filtered water | Xiamen | 270 | B |
| 42 | XM-S2-2 | Activated carbon filtered water | Xiamen | 316 | B |
| 43 | XM-S2-2 | Activated carbon filtered water | Xiamen | 277 | F |
| 44 | BJ-S1-1 | Raw water | Beijing | 274 | B |
| 45 | BJ-S1-1 | Raw water | Beijing | 235 | B |
| 46 | BJ-S1-2 | Activated carbon filtered water | Beijing | 316 | B |
| 47 | BJ-S1-2 | Activated carbon filtered water | Beijing | 1182 | B |
| 48 | BJ-S1-3 | Final water product | Beijing | 1182 | B |
| 49 | BJ-S2-1 | Raw water | Beijing | 1182 | B |
| 50 | BJ-S2-1 | Raw water | Beijing | 357 | B |
| 51 | BJ-S2-2 | Activated carbon filtered water | Beijing | 919 | B |
| 52 | BJ-M3-2 | Activated carbon filtered water | Beijing | 357 | B |
| 53 | BJ-M3-2 | Activated carbon filtered water | Beijing | 316 | B |
| 54 | GZ-S2-1 | Raw water | Guangzhou | 1284 | B |
| 55 | GZ-S2-1 | Raw water | Guangzhou | 313 | B |
| 56 | GZ-S2-2 | Activated carbon filtered water | Guangzhou | 1182 | B |
| 57 | GZ-M3-2 | Activated carbon filtered water | Guangzhou | 313 | B |
| 58 | YF-S4-1 | Raw water | Yunfu | 1239 | B |
| 59 | YF-S5-1 | Raw water | Yunfu | 1239 | B |
| 60 | MZ-S7-2 | Activated carbon filtered water | Meizhou | 699 | B |
| 61 | ZS-M5-1 | Raw water | Zhongshan | 699 | B |
| 62 | ZS-M5-2 | Activated carbon filtered water | Zhongshan | 1094 | B |
| 63 | ZS-M5-2 | Activated carbon filtered water | Zhongshan | 1094 | B |
| 64 | ZS-M5-2 | Activated carbon filtered water | Zhongshan | 277 | F |
| 65 | JM-M15-1A | Raw water | Jiangmen | 111 | B |
| 66 | JM-M15-1B | Raw water | Jiangmen | 111 | B |
| 67 | JM-M15-2B | Raw water | Jiangmen | 111 | B |
| 68 | GZ-S16-1 | Raw water | Guangzhou | 111 | B |
| 69 | GZ-S16-3 | Final water product | Guangzhou | 267 | B |
| 70 | GZ-S16-3 | Final water product | Guangzhou | 267 | B |
| 71 | ZQ-M9-1 | Raw water | Zhaoqing | 313 | B |
| 72 | ZQ-M9-2 | Activated carbon filtered water | Zhaoqing | 267 | B |
| 73 | ZQ-M9-2 | Activated carbon filtered water | Zhaoqing | 835 | B |
| 74 | HY-S8-2 | Activated carbon filtered water | Heyuan | 1967 | F |
| 75 | HY-S9-2 | Activated carbon filtered water | Heyuan | 1311 | F |
| 76 | HY-S9-2 | Activated carbon filtered water | Heyuan | 1967 | F |
| 77 | YJ-S1-2 | Activated carbon filtered water | Yangjiang | 274 | C |
| 78 | YJ-S6-2 | Activated carbon filtered water | Yangjiang | 235 | C |
| 79 | YJ-S7-2 | Activated carbon filtered water | Yangjiang | 235 | C |
| 80 | YJ-S7-2 | Activated carbon filtered water | Yangjiang | 235 | C |
| 81 | MZ-M1-2 | Activated carbon filtered water | Meizhou | 111 | B |
| 82 | MZ-M1-2 | Activated carbon filtered water | Meizhou | 111 | B |
| 83 | JM-S30-1 | Raw water | Jiangmen | 2048 | B |
| 84 | JM-S30-2 | Raw water | Jiangmen | 111 | B |
| 85 | QY-S4-1 | Raw water | Qingyuan | 357 | B |
| 86 | QY-S4-1 | Raw water | Qingyuan | 357 | B |
| 87 | FS-S25-1A | Raw water | Foshan | 244 | B |
| 88 | FS-S25-1A | Raw water | Foshan | 835 | B |
| 89 | FS-S25-1B | Raw water | Foshan | 1973 | B |
| 90 | FS-S25-2 | Activated carbon filtered water | Foshan | 244 | B |
| 91 | FS-S25-2 | Activated carbon filtered water | Foshan | 313 | B |
| 92 | SF-S23-2 | Activated carbon filtered water | Foshan | 1639 | B |
| 93 | DG-M32-2 | Activated carbon filtered water | Dongguan | 2024 | B |
| 94 | DG-M32-2 | Activated carbon filtered water | Dongguan | 111 | B |
| 95 | DG-M32-2 | Activated carbon filtered water | Dongguan | 644 | B |
| 96 | DG-M31-2 | Activated carbon filtered water | Dongguan | 357 | B |
| 97 | DG-M31-2 | Activated carbon filtered water | Dongguan | 1976 | B |
| 98 | DG-M31-2 | Activated carbon filtered water | Dongguan | 639 | B |
| 99 | SG-S7-1A | Raw water | Shaoguan | 175 | B |
| 100 | SG-S7-1A | Raw water | Shaoguan | 175 | B |
| 101 | SG-S7-1B | Raw water | Shaoguan | 235 | B |
| 102 | SG-S7-1B | Raw water | Shaoguan | 235 | B |
| 103 | SG-S7-2 | Activated carbon filtered water | Shaoguan | 773 | B |
| 104 | SG-S7-2 | Activated carbon filtered water | Shaoguan | 882 | B |
| 105 | HZ-S13-1 | Raw water | Huizhou | 1974 | B |
| 106 | HZ-S13-1 | Raw water | Huizhou | 773 | E |
| 107 | HZ-S13-2 | Activated carbon filtered water | Huizhou | 235 | C |
| 108 | HZ-S13-2 | Activated carbon filtered water | Huizhou | 892 | C |
| 109 | HZ-S13-3 | Final water product | Huizhou | 175 | F |
| 110 | HZ-S13-3 | Final water product | Huizhou | 324 | B |
| 111 | ZH-S33-1 | Raw water | Zhuhai | 2133 | B |
| 112 | ZH-S33-2 | Activated carbon filtered water | Zhuhai | 1239 | B |
| 113 | ZH-S33-2 | Activated carbon filtered water | Zhuhai | 235 | F |
| 114 | ZH-S33-2 | Activated carbon filtered water | Zhuhai | 235 | F |
| 115 | ZH-M35-1 | Raw water | Zhuhai | 1239 | C |
| 116 | ZH-M35-2 | Activated carbon filtered water | Zhuhai | 390 | C |
| 117 | GZ-S39-1 | Raw water | Guangzhou | 274 | B |
| 118 | GZ-S39-1 | Raw water | Guangzhou | 274 | B |
| 119 | GZ-S39-1 | Raw water | Guangzhou | 298 | B |
| 120 | GZ-S39-2 | Activated carbon filtered water | Guangzhou | 273 | B |
| 121 | GZ-S39-2 | Activated carbon filtered water | Guangzhou | 273 | B |
| 122 | GZ-S39-2 | Activated carbon filtered water | Guangzhou | 298 | B |
| 123 | GZ-S42-1 | Raw water | Guangzhou | 298 | B |
| 124 | GZ-S42-1 | Raw water | Guangzhou | 273 | B |
| 125 | GZ-S42-1 | Raw water | Guangzhou | 463 | E |
| 126 | GZ-S42-2 | Activated carbon filtered water | Guangzhou | 298 | B |
| 127 | GZ-S42-2 | Activated carbon filtered water | Guangzhou | 644 | E |
| 128 | GZ-S42-2 | Activated carbon filtered water | Guangzhou | 644 | E |
| 129 | GZ-S42-2 | Activated carbon filtered water | Guangzhou | 2106 | B |
| 130 | GZ-S42-2 | Activated carbon filtered water | Guangzhou | 463 | B |
| 131 | GZ-S42-3 | Final water product | Guangzhou | 298 | B |
| 132 | GZ-S42-3 | Final water product | Guangzhou | 298 | B |

**2. Supplementary Figures**

**
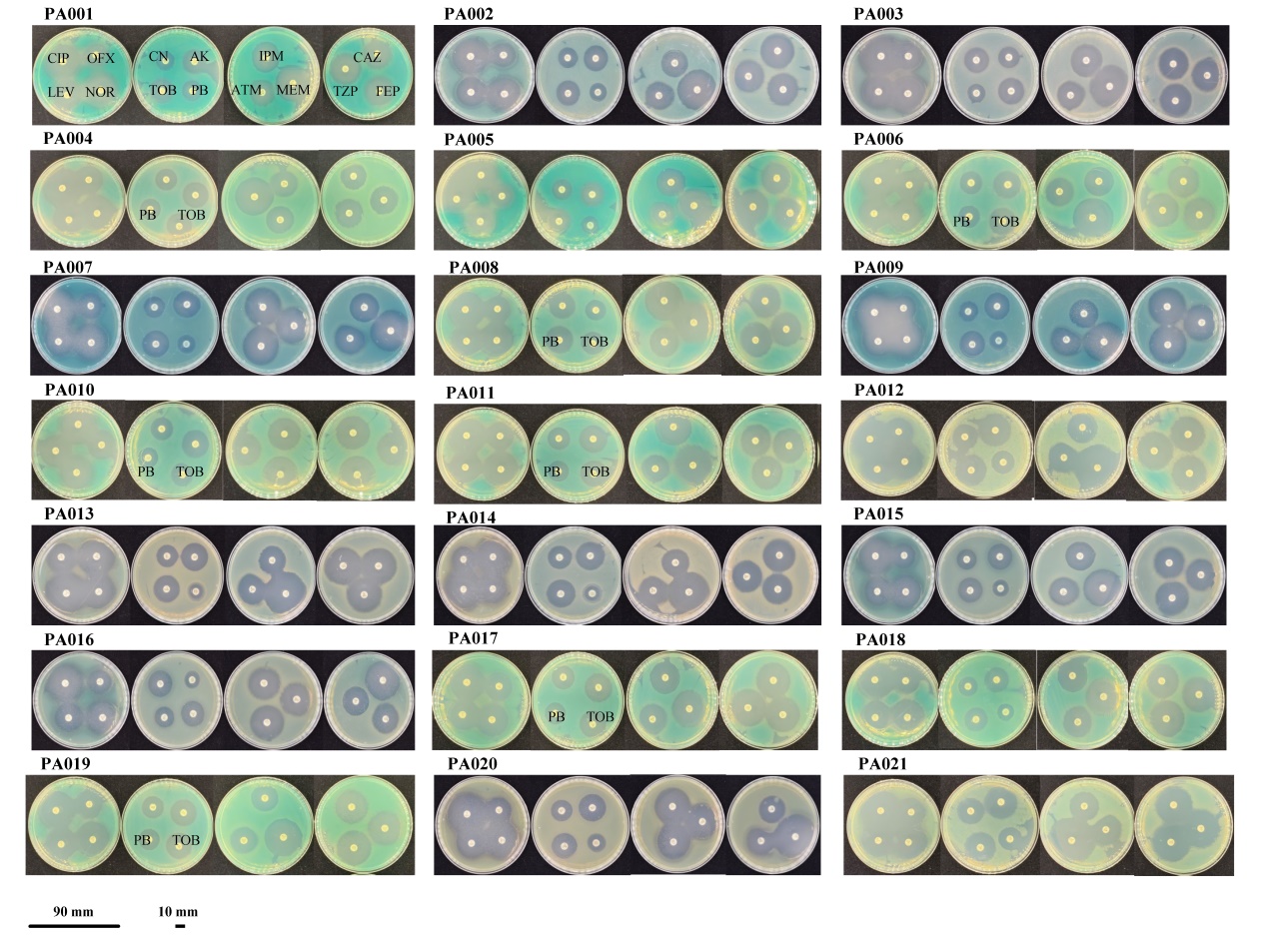
**

**
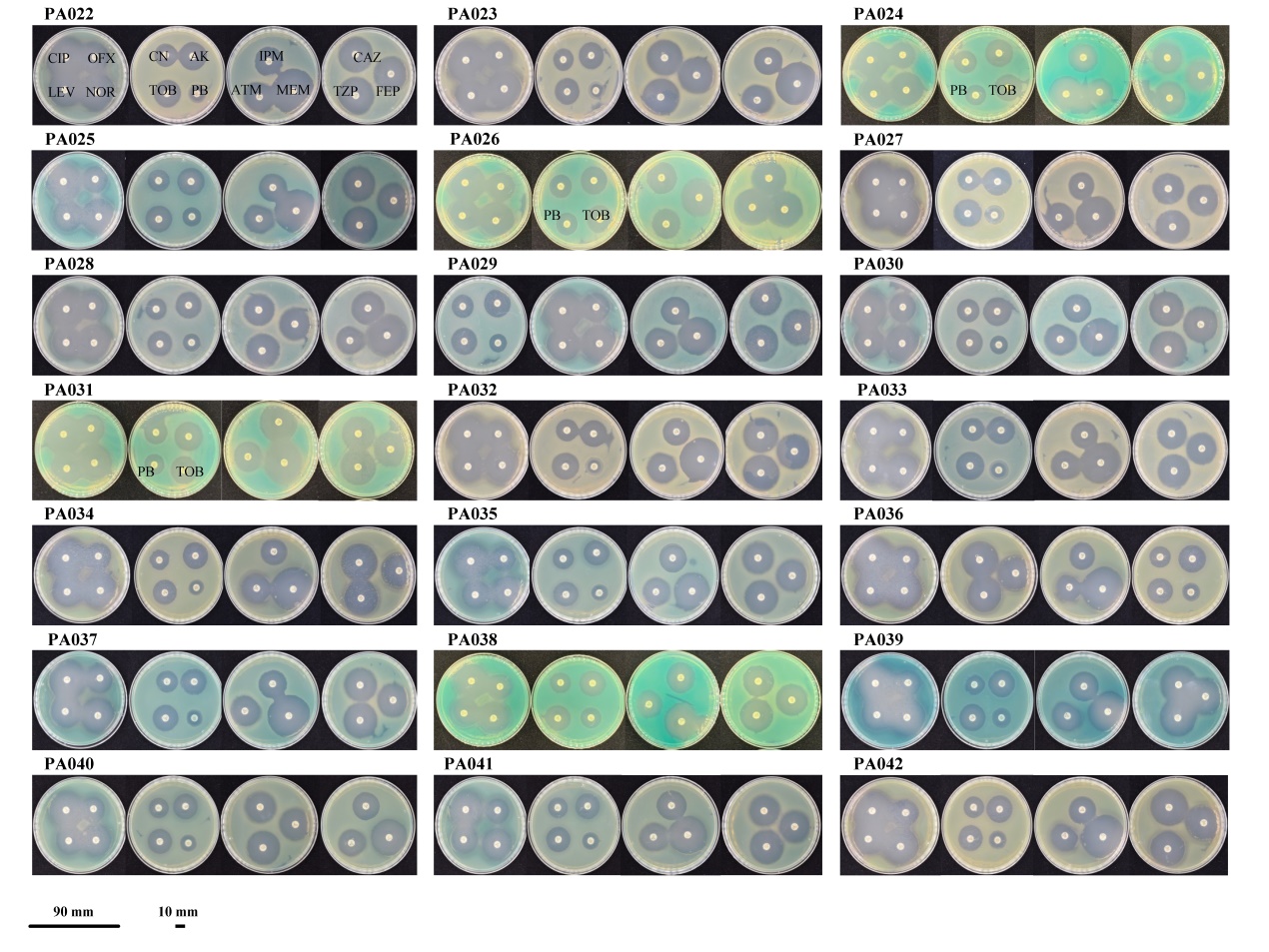
**


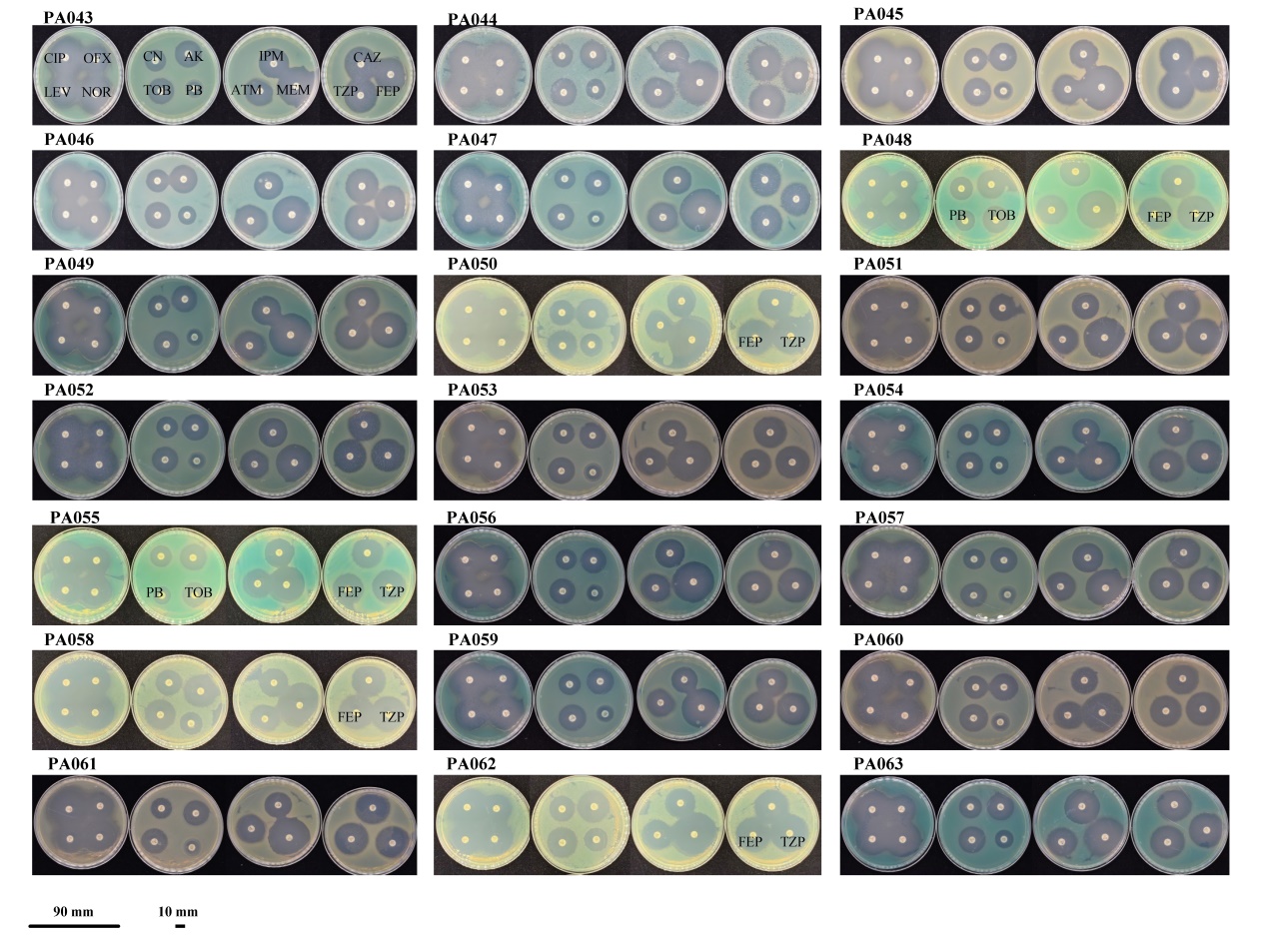


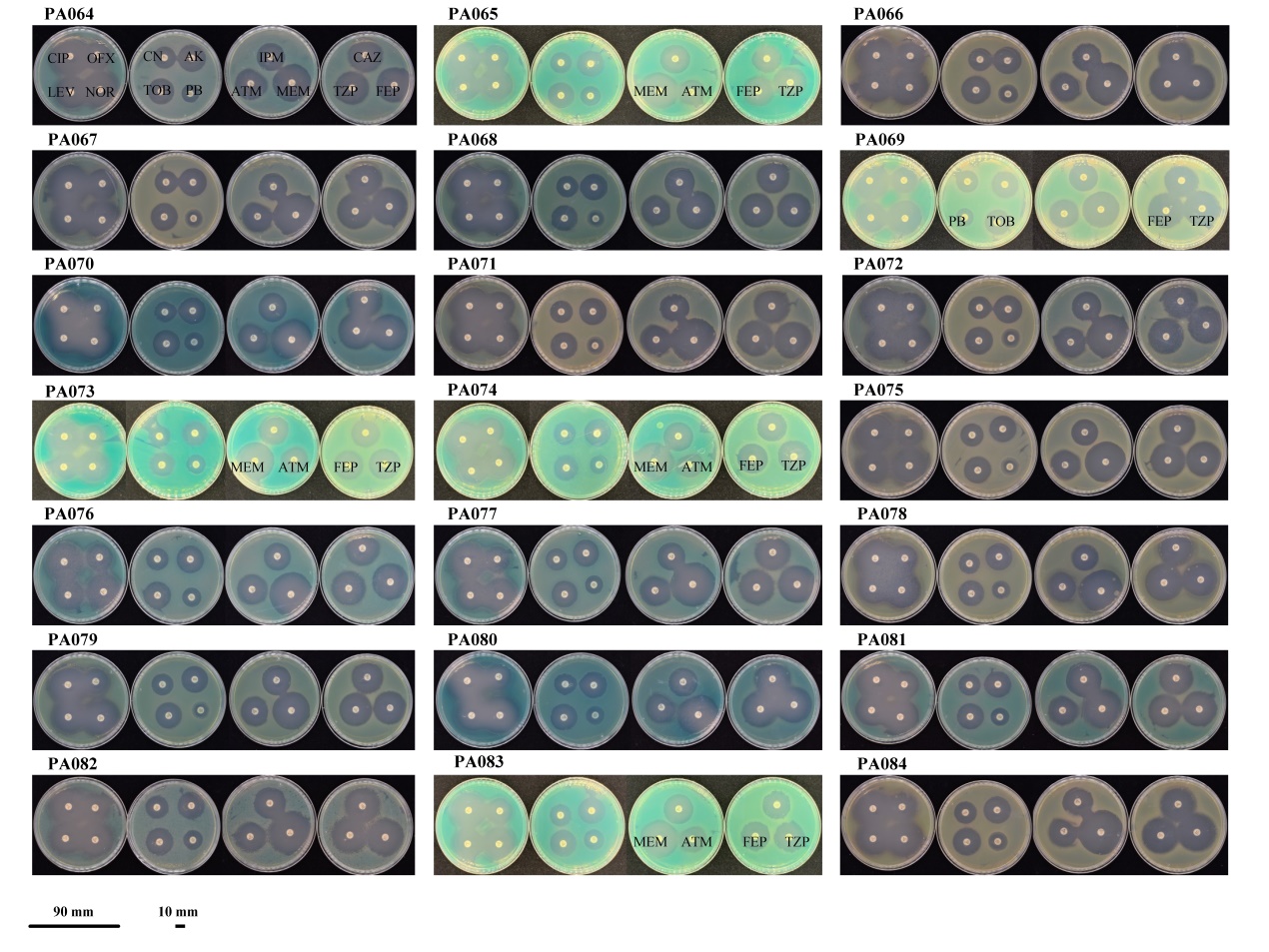


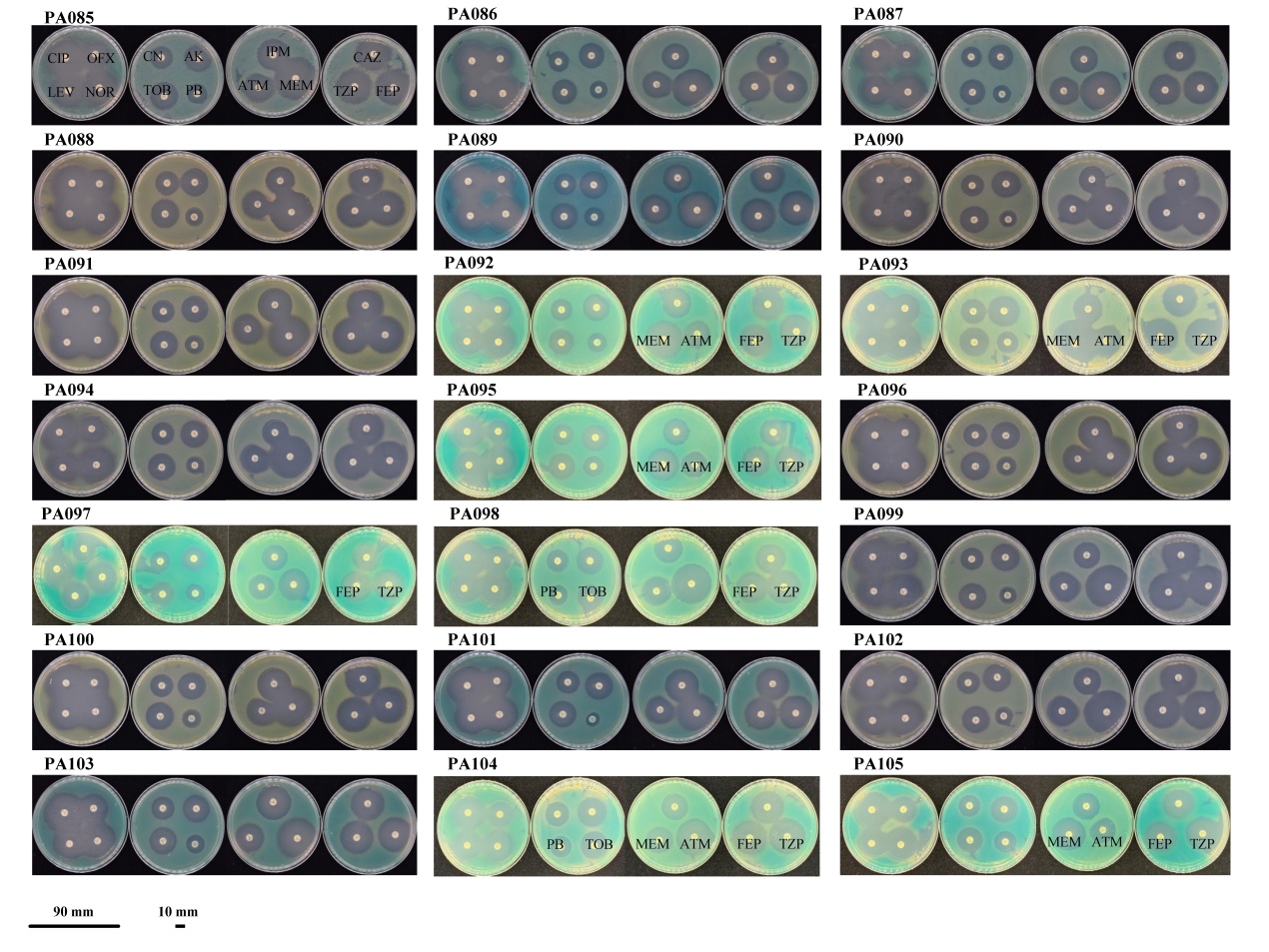


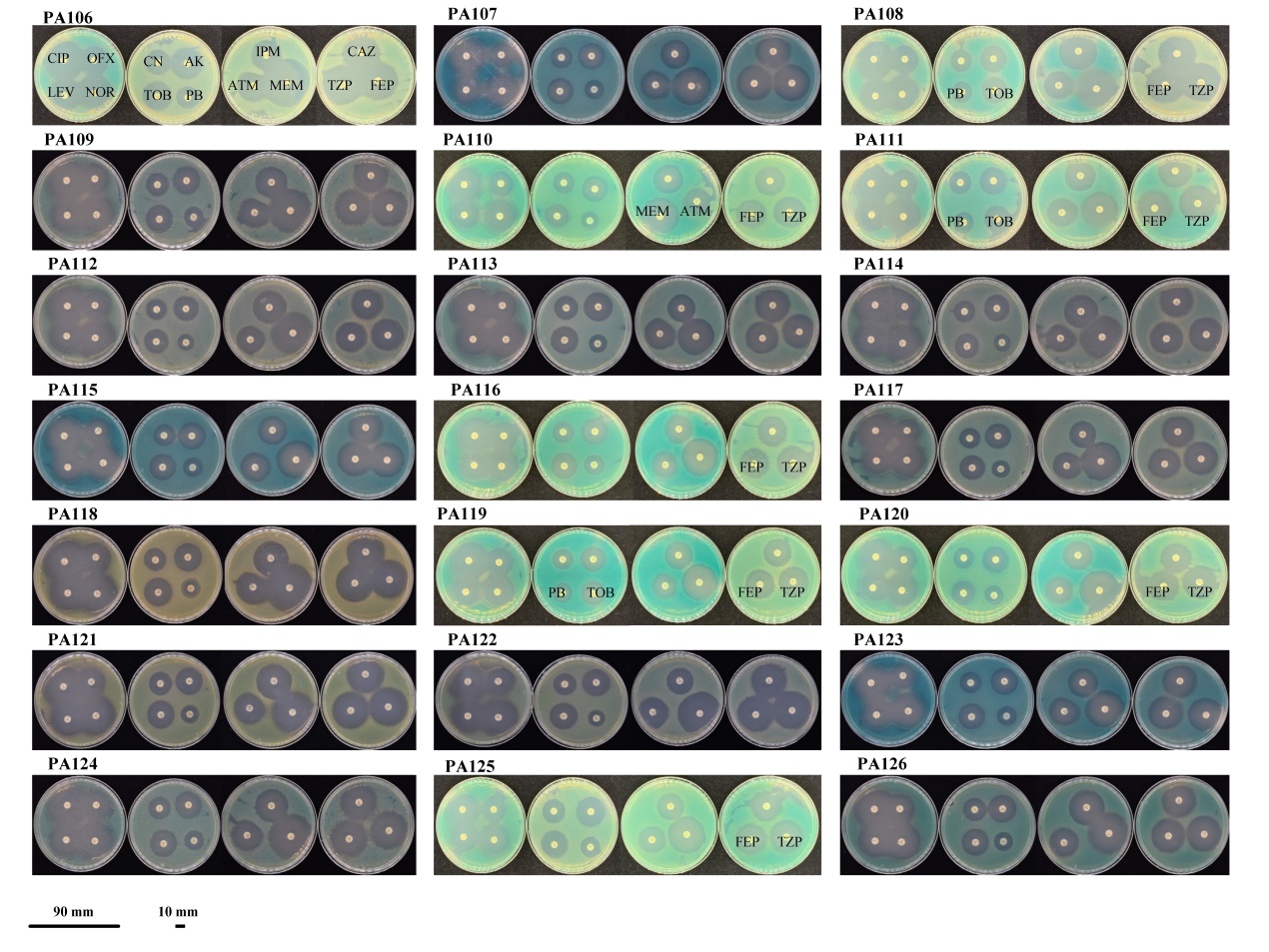


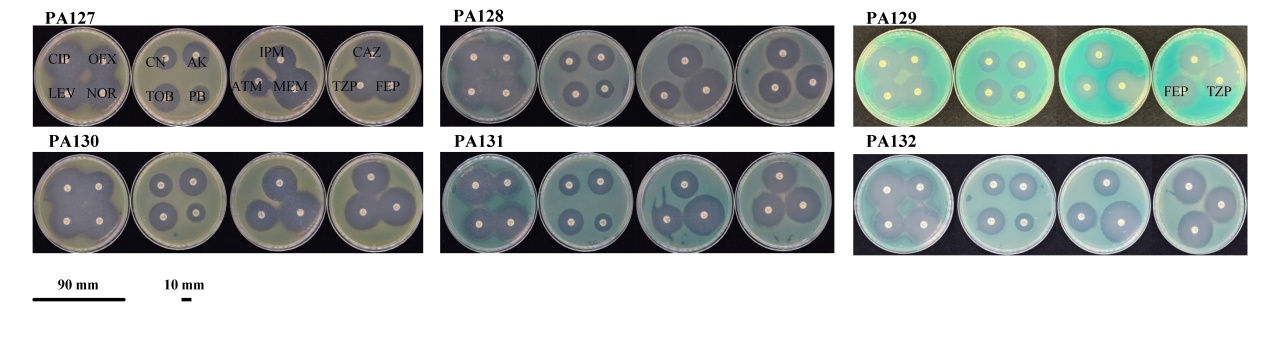


FIGURE S1 Antibiotic resistance of *P. aeruginosa* isolates examined in this study.

^
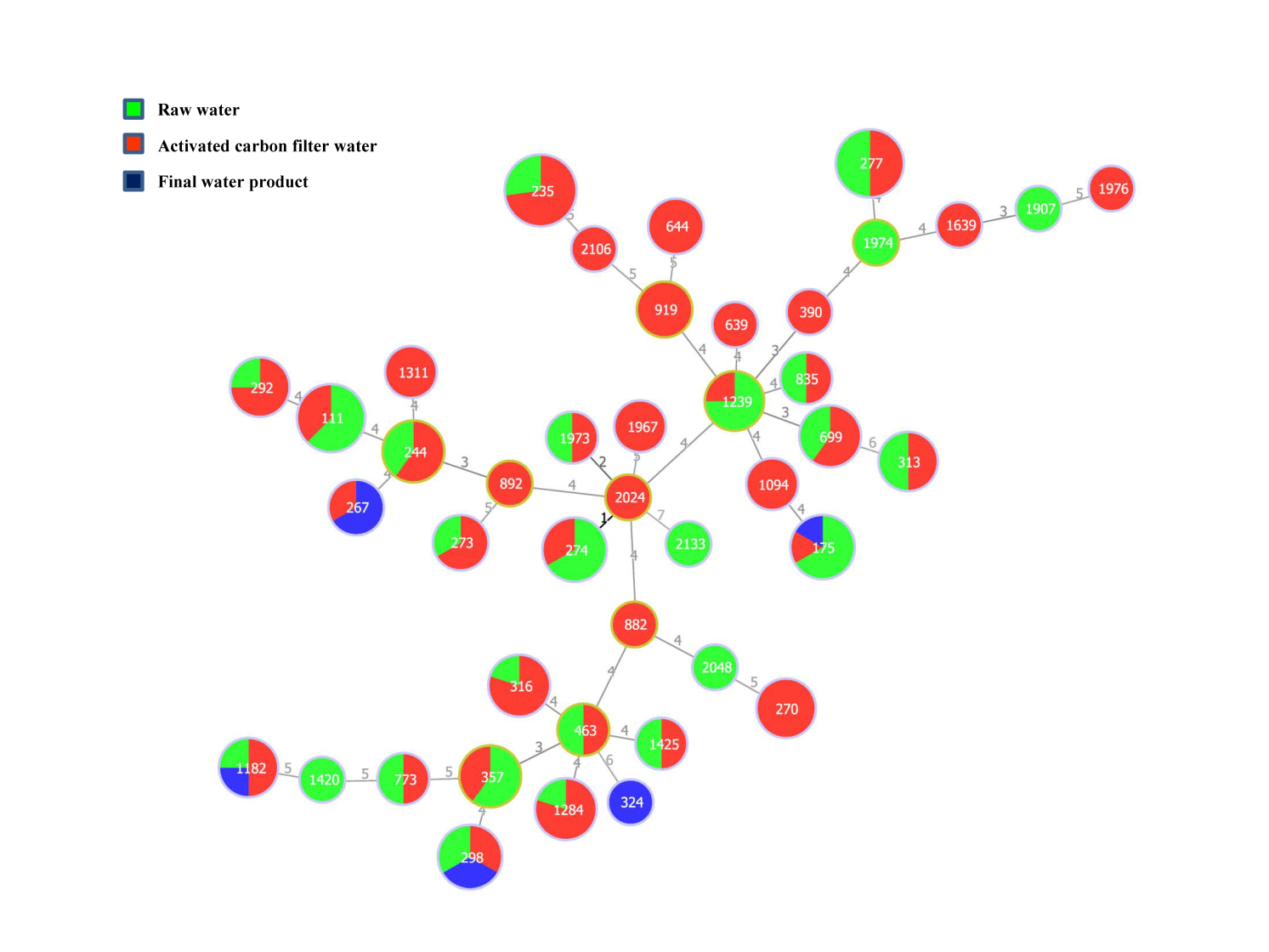
^FIGURE S2 Minimum spanning tree based on multilocus sequence typing data for the 132 *P. aeruginosa* isolates examined in this study. Each circle represents one ST. The size of circle is related to the number of strains within this ST. The colors in the circles represent the sample types.
